# Characterization of lipomatous tumors with high-resolution ^1^H MRS at 17.6T: Do benign lipomas, atypical lipomatous tumors and liposarcomas have a distinct metabolic signature?

**DOI:** 10.3389/fonc.2022.920560

**Published:** 2022-09-09

**Authors:** Santosh Kumar Bharti, Brett A. Shannon, Raj Kumar Sharma, Adam S. Levin, Carol D. Morris, Zaver M. Bhujwalla, Laura M. Fayad

**Affiliations:** ^1^ Division of Cancer Imaging Research, The Russell H. Morgan Department of Radiology and Radiological Science, The Johns Hopkins University School of Medicine, Baltimore, MD, United States; ^2^ Department of Orthopaedic Surgery, The Johns Hopkins University School of Medicine, Baltimore, MD, United States; ^3^ Sidney Kimmel Comprehensive Cancer Center, The Johns Hopkins University School of Medicine, Baltimore, MD, United States; ^4^ Department of Radiation Oncology and Molecular Radiation Sciences, The Johns Hopkins University School of Medicine, Baltimore, MD, United States; ^5^ Musculoskeletal Radiology, The Russell H. Morgan Department of Radiology and Radiological Science; The Johns Hopkins University School of Medicine, Baltimore, MD, United States

**Keywords:** lipoma, liposarcoma, atypical lipomatous tumor, ^1^H MR spectroscopy, metabolites

## Abstract

**Background:**

Distinguishing between some benign lipomas (BLs), atypical lipomatous tumors (ALTs), and dedifferentiated liposarcomas (DDLs) can be challenging due to overlapping magnetic resonance imaging characteristics, and poorly understood molecular mechanisms underlying the malignant transformation of liposarcomas.

**Purpose:**

To identify metabolic biomarkers of the lipomatous tumor spectrum by examining human tissue specimens using high-resolution ^1^H magnetic resonance spectroscopy (MRS).

**Materials and methods:**

In this prospective study, human tissue specimens were obtained from participants who underwent surgical resection for radiologically-indeterminate lipomatous tumors between November 2016 and May 2019. Tissue specimens were obtained from normal subcutaneous fat (n=9), BLs (n=10), ALTs (n=7) and DDLs (n=8). Extracts from specimens were examined with high-resolution MRS at 17.6T. Computational modeling of pattern recognition-based cluster analysis was utilized to identify significant differences in metabolic signatures between the lipomatous tumor types.

**Results:**

Significant differences between BLs and ALTs were observed for multiple metabolites, including leucine, valine, branched chain amino acids, alanine, acetate, glutamine, and formate. DDLs were distinguished from ALTs by increased glucose and lactate, and increased phosphatidylcholine. Multivariate principal component analysis showed clear clustering identifying distinct metabolic signatures of the tissue types.

**Conclusion:**

Metabolic signatures identified in ^1^H MR spectra of lipomatous tumors provide new insights into malignant progression and metabolic targeting. The metabolic patterns identified provide the foundation of developing noninvasive MRS or PET imaging biomarkers to distinguish between BLs, ALTs, and DDLs.

## Highlights

1. Proton MRS demonstrates distinct metabolic signatures of benign lipomas, atypical lipomatous tumors, and dedifferentiated liposarcomas.
2. The aqueous-phase metabolite profile of atypical lipomatous tumors appears more similar to that of dedifferentiated liposarcomas than to that of benign lipomas.
3. Dedifferentiated liposarcomas may be distinguished from atypical lipomatous tumors by increased glucose and lactate.

## Introduction

Lipomatous tumors are categorized as malignant (liposarcomas), benign (simple lipomas and numerous variants), or intermediate-grade (atypical lipomatous tumors [ALTs], also known as well-differentiated liposarcomas [WDLs]) (1). ALT/WDLs are associated with local recurrence after resection (2–4), and have the potential for malignant transformation to dedifferentiated liposarcoma (DDL), a process that is poorly understood. ALT/WDLs and DDLs share a characteristic 12q13-15 chromosomal amplification, which encodes several oncogenes, including the p53 antagonist MDM2, CDK4, and p16 (5, 6) that are absent in normal fat and benign lipomas (BLs). ALTs located in an extremity may be observed with serial imaging or marginally excised, depending upon the preferences of the patient and judgement of the treating surgeon. However, tumors near or deep to important anatomic structures may present a clinical dilemma due to the risk of neurologic, vascular, or functional impairment in the setting of this low but uncertain risk of malignant oncologic transformation. Thus, accurate characterization is critical to guide treatment, but some lipomas, ALT/WDLs, and DDLs have similar characteristics on magnetic resonance imaging (MRI). Differentiating these entities can therefore be challenging (7–9), with one study reporting diagnostic accuracy of 69% for differentiating lipomas from ALT/WDLs (10). Such limited accuracy of imaging has led some to conclude that the diagnosis should be based on assessment of molecular features (11).

MR spectroscopy (MRS) presents an opportunity for noninvasive metabolic tumor characterization, including identification of metabolic signatures and targeted analysis of specific pathways. Previously, the feasibility of measuring choline, a component of cell membrane phospholipids, by ^1^H MRS at 3T has been demonstrated in musculoskeletal lesions (12). Abnormal choline metabolism is one of the most consistent features of cancer (13). A systematic review of investigations using qualitative and semiquantitative ^1^H MRS techniques revealed significantly elevated total choline levels in malignant musculoskeletal tumors compared with their benign counterparts (14). MRS has been used to correlate abnormal phosphatidylcholine metabolism with cancer aggressiveness (15, 16) and this technique is improved by higher magnetic field strength.

Metabolomics with high-resolution ^1^H MRS of excised tissues performed at high field strengths has significant potential for disease biomarker screening, pathological mechanism interpretation, drug efficacy evaluation as well as altered metabolic pathways/flux assessment (17, 18). The hypothesis of this study is that metabolomics with high-resolution ^1^H MRS provides discriminatory features for characterizing the spectrum of lipomatous tumors. The purpose of this study was to identify metabolic determinants for lipomatous tumor classification using high-resolution ^1^H MRS at 17.6T, with the goal of developing metabolic signatures for benign, intermediate-grade and high-grade tumors. This information can help elucidate the underlying metabolic pathways of pathogenesis and dedifferentiation.

## Methods and materials

### Overall study design

This study was approved by the Johns Hopkins University School of Medicine eIRB2 institutional review board. High-resolution ^1^H MRS was performed on surgical specimens obtained from participants with indeterminate lipomatous tumors, at 17.6T. Computational modeling of pattern recognition-based cluster analysis of the metabolomic data was utilized to identify significant differences in metabolic signatures between benign, intermediate grade and high grade lipomatous tumors.

### Patient’s selection, inclusion & exclusion criteria

Subjects were recruited at a single referral institution at the time of presentation to orthopedic oncology clinic. Details of age, gender, and pathology are provided in **Table 1**. Inclusion criteria were patients with lipomatous tumors who were deemed to have indeterminate lesions by clinical and MRI features and who underwent surgical excision between November 2016 and May 2019. Exclusion criteria were patients with liposarcomas other than DDL, those with prior surgical or pharmacologic treatment, and patients who refused to provide surgical specimens. The main clinical problem being addressed by this study is the differentiation of BL, ALT and DDL. The imaging and clinical features of these entities (BL, ALT and DDL) overlap and, in particular, a de-differentiated liposarcoma has imaging features in common with ALT rather than other forms of liposarcoma. Other liposarcoma types have very different imaging characteristics and would not be confused with ALT or BL. Therefore, the study was focused around characterizing BL, ALT and DDL, with the exclusion of other forms of liposarcoma.

**Table 1:** Patient age, sex, associated pathology, and lesion size.

Lipoma			
Age	Sex	Size (cm)	Normal Fat Sampled
47	F	11	Y
37	F	8	Y
70	M	3	
37	F	9	Y
46	M	10	
63	M	17	
31	F	15	
59	F	12	
63	F	13	
68	F	25	
**Atypical Lipomatous Tumor**			
51	F	8	Y
55	M	20	Y
65	F	23	Y
58	F	17	
70	F	17	Y
66	F	26	
87	F	30	
**Dedifferentiated Liposarcoma**			
54	M	20	Y
67	M	18	Y
69	M	17	
68	F	9	
42	M	16	
66	M	27	
82	M	13	
63	F	14	

Specimens from a total of 25 participants were included in this study. The study was performed with 10 BL, 7 ALT/WDL, 8 DDL, and 9 normal subcutaneous fat specimens. The normal fat specimens were collected from patients with BL (3), ALT/WDL (4), and DDL (2). The normal fat specimens had a fairly homogenous metabolic profile that was similar to BL and distinguishable from ALT/WDL and DDL.

Tissue specimens were acquired from upper and lower extremities and the retroperitoneum in participants. Eligible patients included in the study had their tumor (either BL, ALT, or DDLS) surgically excised. Following operative excision, the tumor was incised away from the operative field, and a sample was harvested under sterile conditions from the tumor for analysis. Three subjects were excluded after they were found to have sarcomas other than DDL (two myxofibrosarcomas and one pleomorphic liposarcoma).

### Histologic assessment

Tumors were removed in their entirety during surgery and taken immediately to the surgical pathology lab. Gross and histologic assessment was performed by one of two experienced musculoskeletal pathologists. According to routine protocol, MDM2 staining or amplification was performed for specimens with increased cellularity, any atypia, or other concerning features. The pathologists’ diagnosis was used to determine tumor type.

### Tissue sample acquisition and processing

During routine gross pathologic assessment, representative sections of tissue of approximately 1 cc in size were dissected from the central portions of the BLs, ALTs and DDLs. While central necrosis can be seen in some sarcomas, this was not a prominent feature in any of the tumors excised in the current study. Following excision, each tumor was evaluated with standard histopathology, including necrosis, and none demonstrated any significant spontaneous necrosis. An approximately 1 cc sample of non-involved normal subcutaneous fat tissue encountered in surgical approach was also excised. Tissues were immediately snap frozen in liquid nitrogen and stored at -80°C. Whole snap frozen tissue samples were powdered under liquid nitrogen, weighed, and dual phase solvent extraction was performed using methanol/chloroform/water (2:1:1). Approximately 300 mg powdered tissue sample was taken and suspended in 4 mL ice-cold methanol, vigorously vortexed, and kept on ice for 10 minutes. Next, 8 mL of chloroform was added, and the solution was vortexed and sonicated for 2 minutes under ice-cold conditions at 1 s pulse intervals. After this, 4 mL of water was added, and the sample was thoroughly mixed and kept overnight for phase separation at 4°C. Samples were centrifuged for 30 minutes at 4000 g at 4°C to separate the phases. The top aqueous phase was collected, evaporated under a stream of nitrogen to evaporate methanol, and later lyophilized to remove the remaining water. The lipid phase was kept under a stream of nitrogen to evaporate chloroform and methanol. Samples were reconstituted in 650 μL of 1X phosphate buffered D_2_O (90% D_2_O, 10% H_2_O, pH = 7.4) with trimethylsilyl propionic acid sodium salt (TSP), vortexed, centrifuged at 4000 g for 5 min at 4°C, and supernatants were analyzed with ^1^H MRS. The lipid phase was dried under a stream of nitrogen, the residue dissolved in 500 µl deuterated chloroform and methanol (2:1), and the preparation transferred to nuclear magnetic resonance (NMR) tubes for MRS analysis.

### ^1^H MRS analysis of tumor extracts

High-resolution proton MR spectra were acquired at room temperature on a Bruker Avance III 750 MHz (17.6 T) MR spectrometer equipped with a 5 mm probe. Spectra of aqueous-phase sample preparations with water suppression were acquired using water pre-saturation and a single pulse sequence with the following parameters: spectral width of 15495.86 Hz, data points of 64 K, 90° flip angle, relaxation delay of 10 sec, acquisition time 2.11 sec, 64 scans with 8 dummy scans, receiver gain 64. Lipid-phase samples were acquired with the same parameters except that the number of scans was reduced to 16. Spectral acquisition, processing and quantification were performed using TOPSPIN 3.5 software. Characterization of the aqueous- and lipid-phase metabolites was performed based on chemical shift, coupling constant, and the splitting pattern of metabolites as reported in literature using standard MR spectra of metabolites from the Biological Magnetic Resonance Bank (19) and two-dimensional NMR methods (20). Area under peaks were integrated and normalized with respect to TSP used as a chemical shift and concentration standard, and the tissue weight used to prepare the sample (21). Characterization of the lipid-phase spectra was performed using TMS as a chemical shift reference (22). Since TMS is highly volatile the artificial signal from TOPSPIN was used for normalization.

### Heat maps and principal component analysis

Metabolic heat maps were generated from quantitative analysis of high-resolution one-dimensional ^1^H MR spectral data from the aqueous-phase metabolites using MATLAB software (MATLAB R2017b, MathWorks) to visualize the metabolic patterns. Due to the high dynamic range of metabolites, we normalized the highest intensity of a particular metabolite in any of the three groups to 100%. This normalization provided a dynamic range between 0 - 100%, allowing a better presentation of the heat map that represents average measurements of multiple replicates per group. The integral area under the peak was normalized to weight and volume of the sample. TSP dissolved in D_2_O was used as a quantitative reference in the spectral analysis.

To investigate the change in overall metabolic pattern in normal fat, BLs, ALTs and DDLs, multivariate principal component analysis (PCA) was performed on the quantitative data from aqueous phase metabolites. Using Bruker AMIX software, selective variable size binning method was used to quantify the non-overlapping peaks in ^1^H MR spectra, and broad water resonance at 4.7 ppm and other overlapping peaks were excluded from the analysis. Integral peak areas were normalized to the reference TSP peak and tissue weight.

### Statistical analysis

Graphpad Prism 8.0 software was used. Metabolite levels in the four specimen groups (normal subcutaneous fat, BLs, ALTs/WDLs, DDLs) were compared using a One Way ANOVA with a Tukey Test correction for multiple comparisons, with a corrected *p*-value ≤ 0.05 considered significant.

## Results

### General metabolic profile of lipomatous tumors

**Figure 1:**
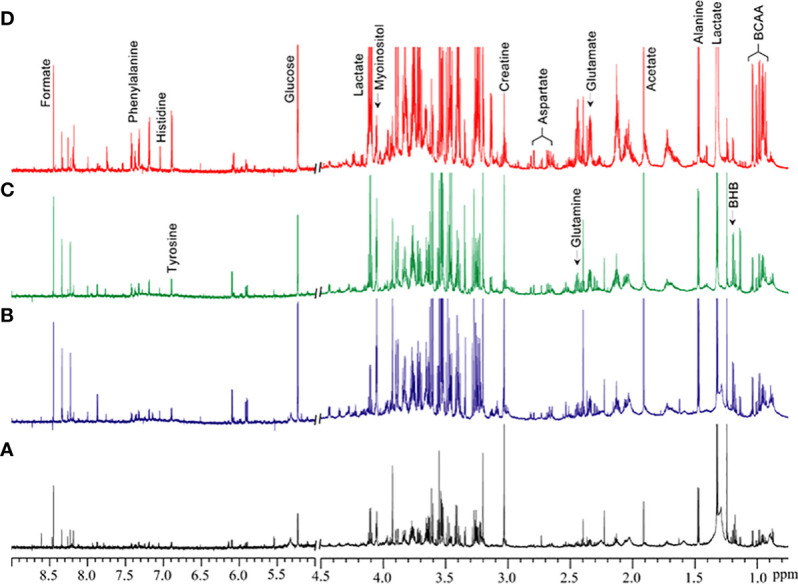
Representative ^1^H MR spectra showing metabolic differences in aqueous-phase extracts of tumor tissue obtained from **(A)** normal fat, **(B)** benign lipoma (BL), **(C)** atypical lipomatous tumor (ALT), and **(D)** dedifferentiated liposarcoma (DDL). Normal fat tissues are non-involved tissues obtained during tumor excision surgery. BCAA, branched chain amino acids; BHB, beta-hydroxybutyrate.

**Figure 1** shows the metabolic information obtained from aqueous-phase spectra of normal subcutaneous fat and of the different lipomatous tumor types analyzed. Aqueous-phase spectra with the TSP reference included are presented in **Supplementary Figure 1**. We quantified the metabolites to evaluate the ability of ^1^H MRS to discriminate between tumors and normal fat tissue, based on general metabolic features.

### ^1^H MR spectroscopic analysis and quantification of tumor metabolites

**Figure 2:**
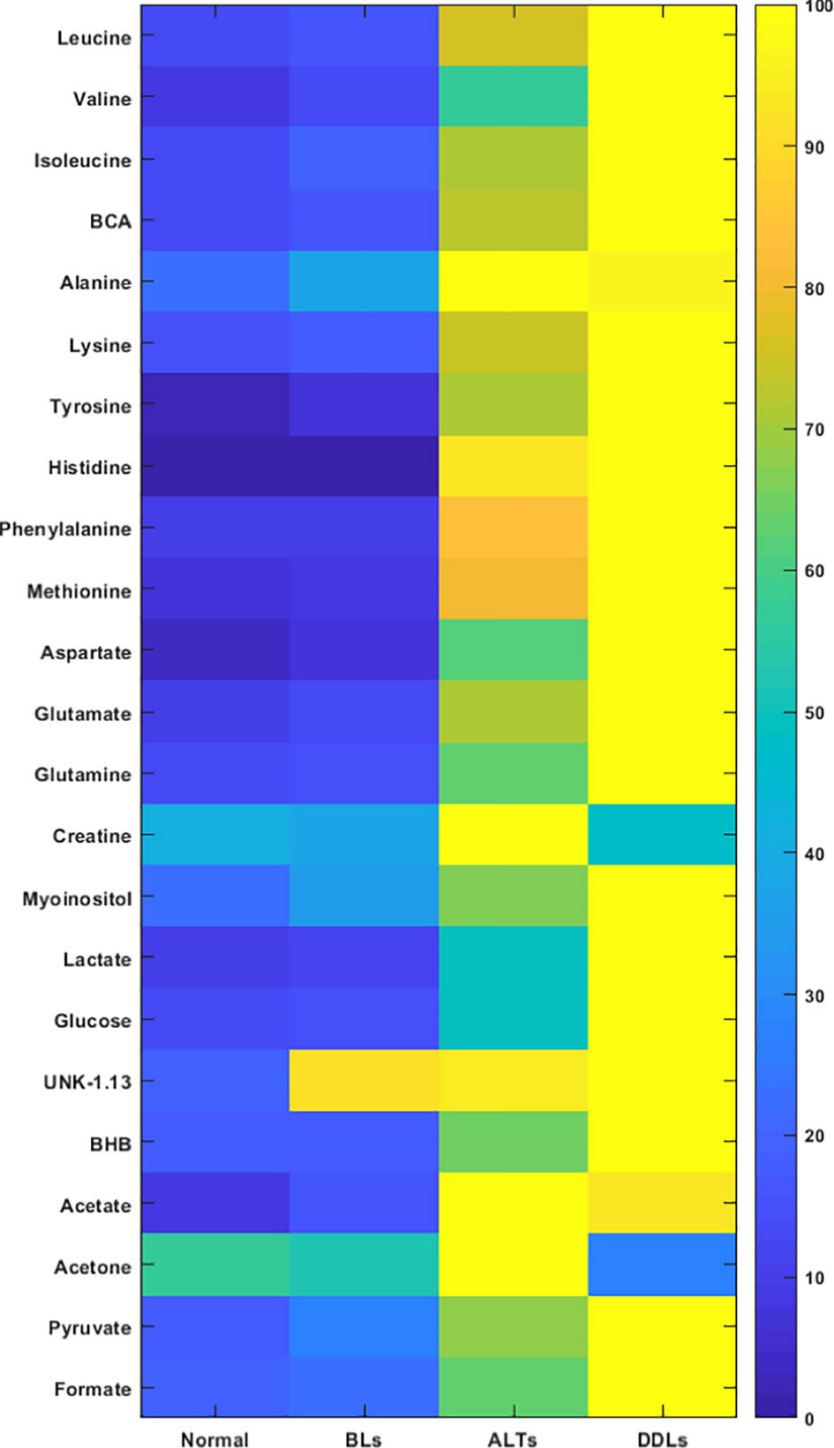
Metabolic heat map displaying differences in the metabolic profile of normal fat (n = 9), benign lipoma (BLs, n = 10), atypical lipomatous tumor (ALTs, n = 7), and dedifferentiated liposarcoma (DDLs, n = 8). Statistical analysis for the different groups can be found in **Table 2**.

Quantitative metabolic data of the differences in metabolites are presented in **Table 2**. As shown in **Table 2**, metabolites that were identified and quantified from the aqueous-phase fractions of tumors were: leucine, valine, isoleucine, branched chain amino acids (BCAA, the sum of leucine, isoleucine, and valine), beta-hydroxybutyrate (BHB), alanine, lysine, acetate, acetone, glutamate, pyruvate, glutamine, methionine, aspartate, creatine, myoinositol, lactate, glucose, tyrosine, histidine, phenylalanine, formate. No significant differences were identified in the levels of any of the metabolites when comparing normal fat tissue with BLs. However, with p-values shown in brackets, leucine (0.05), valine (0.04), BCAA (leucine + isoleucine + valine, (0.05)), alanine (0.03), acetate (0.04), glutamine (0.04), formate (0.02), glucose (0.05) and lactate (0.05) were significantly increased in ALTs compared with normal fat tissue. Similarly, leucine (0.03), valine (0.04), BCAA (0.008), acetate (0.03), glutamate (0.009), glutamine (0.03), lactate (0.03), glucose (0.04), phenylalanine (0.04) and formate (0.01) were significantly higher in ALTs tumors compared to BLs. Large metabolic differences were observed between BLs and DDLs, with significant changes in leucine (0.01), valine (0.01), isoleucine (0.01), BCAA (0.01), BHB (0.04), lysine (0.02), glutamate (0.01), pyruvate (0.02), glutamine (0.01), methionine (0.04), aspartate (0.01), myoinositol (0.01), lactate (0.01), glucose (0.01), tyrosine (0.02), phenylalanine (0.04), and formate (0.01). A heat map displaying the pattern of metabolic differences in the four groups shown in **Figure 2** provides an overview of the metabolic differences between normal fat tissue and BLs, ALT/WDLs and DDLs, and demonstrates that most metabolic changes gradually increased from normal fat to BLs, ALTs and DDLs. Furthermore, lactate (0.02) and glucose (0.009) significantly increased almost two-fold in DDLs compared to ALTs, as well as compared to normal tissue and BLs. These data are summarized as bar plots in **Supplementary Figure 2**.

**Table 2:** List of metabolites quantified from the aqueous-phase of normal fat (n = 9), benign lipoma (BLs, n=10), well-differentiated liposarcoma (ALTs/WDL, n=7), and malignant grade dedifferentiated liposarcoma (DDLs, n=8).

Metabolite name	Chem.Shift ppm	Normal vs BLs *(p-value)*	Normal vs ALTs *(p-value)*	Normalvs DDLs *(p-value)*	BLs vs ALTs *(p-value)*	BLs vs DDLs *(p-value)*	ALTs vs DDLs*(p-value)*	Metabolite Concentration (Mean and SE)							
								Normal		BLs		ALTs		DDLs	
								Mean	SE	Mean	SE	Mean	SE	Mean	SE
**Leucine**	0.95	0.9992	**0.0479**	**0.0147**	**0.0318**	**0.0102**	0.7956	0.1093	0.0138	0.1335	0.0397	0.6318	0.2119	0.8315	0.2143
**Valine**	1.00	0.9925	**0.0444**	**0.0005**	**0.0420**	**0.0003**	0.1516	0.0193	0.0029	0.0306	0.0109	0.1374	0.0445	0.2428	0.0547
**Isoleucine**	1.03	0.9891	0.0593	**0.0110**	0.0634	**0.0113**	0.6829	0.0477	0.0071	0.0724	0.0210	0.2651	0.0774	0.3696	0.1273
**BCAA**	0.98	0.9981	**0.0537**	**0.0114**	**0.0404**	**0.0085**	0.7147	0.3063	0.0401	0.3924	0.1216	1.6889	0.5318	2.3114	0.6866
**UNK-1.13**	1.13	0.5071	0.5102	0.5833	0.9999	0.9987	0.9997	0.0348	0.0054	0.1634	0.0834	0.1696	0.0386	0.1794	0.0875
**BHB**	1.19	0.9999	0.3136	0.0595	0.2277	**0.0372**	0.6511	0.1233	0.0328	0.1244	0.0530	0.4405	0.1433	0.6793	0.3166
**Alanine**	1.47	0.9186	**0.0301**	0.1036	0.0664	0.2112	0.9993	0.1677	0.0199	0.2909	0.0611	0.7575	0.2092	0.7286	0.2451
**Lysine**	1.68	0.9975	0.0799	**0.0215**	0.0663	**0.0177**	0.7706	0.2395	0.0300	0.3126	0.0986	1.2462	0.3966	1.6875	0.5821
**Acetate**	1.91	0.9940	**0.0392**	0.1453	**0.0346**	0.1594	0.9970	0.0457	0.0055	0.0849	0.0353	0.4966	0.1854	0.4596	0.1430
**Acetone**	2.23	0.9986	0.4730	0.8177	0.2929	0.8509	0.1427	0.3677	0.1116	0.3390	0.1272	0.6451	0.1487	0.1724	0.0770
**Glutamate**	2.34	0.9981	0.0581	**0.0121**	**0.0441**	**0.0090**	0.7088	0.0804	0.0132	0.1119	0.0300	0.5843	0.1962	0.8163	0.2674
**Pyruvate**	2.39	0.9586	0.1087	**0.0134**	0.1804	**0.0211**	0.5769	0.0534	0.0113	0.0844	0.0211	0.2059	0.0563	0.3008	0.1033
**Glutamine**	2.44	0.9995	**0.0427**	**0.0012**	**0.0265**	**0.0006**	0.2842	0.1046	0.0177	0.1202	0.0315	0.5049	0.1462	0.8016	0.1992
**Methionine**	2.64	0.9999	0.0926	0.0621	0.0570	**0.0430**	0.9431	0.0211	0.0046	0.0255	0.0113	0.2278	0.0904	0.2819	0.1130
**Aspartate**	2.80	0.9995	0.0982	**0.0086**	0.0701	**0.0054**	0.4965	0.0103	0.0047	0.0148	0.0088	0.1357	0.0498	0.2189	0.0804
**Creatine**	3.03	0.9995	0.2446	0.9984	0.1306	0.9923	0.4399	0.2100	0.0404	0.1929	0.0246	0.5096	0.1902	0.2405	0.0829
**Myoinositol**	4.05	0.9070	0.1044	**0.0061**	0.2397	**0.0138**	0.4042	0.2253	0.0323	0.3391	0.0465	0.6449	0.1097	0.9620	0.3588
**Lactate**	4.11	0.9990	**0.0498**	**0.0001**	**0.0342**	**0.0001**	**0.0229**	0.2214	0.0303	0.2646	0.0381	1.0842	0.2249	2.1760	0.6422
**Glucose**	5.23	0.9993	**0.0541**	**0.0001**	**0.0361**	**0.0001**	**0.0093**	0.0636	0.0058	0.0713	0.0224	0.2358	0.0624	0.4824	0.0867
**Tyrosine**	6.88	0.9955	0.0715	**0.0207**	0.0645	**0.0187**	0.7868	0.0021	0.0023	0.0065	0.0053	0.0666	0.0261	0.0928	0.0318
**Histidine**	7.04	0.9985	0.1296	0.2006	0.1084	0.1934	0.9996	0.0051	0.0011	0.0050	0.0057	0.0660	0.0329	0.0712	0.0390
**Phenylalanine**	7.42	0.9999	0.0808	0.0613	**0.0400**	**0.0361**	0.9564	0.0064	0.0021	0.0059	0.0027	0.0508	0.0205	0.0610	0.0168
**Formate**	8.45	0.9977	**0.0170**	**0.0001**	**0.0111**	**0.0001**	0.0888	0.0155	0.0025	0.0173	0.0053	0.0487	0.0088	0.0778	0.0140
**Glutamine/Glutamate**	NA	0.7250	0.5308	0.9999	0.9760	0.8098	0.6410	1.3275	0.1636	1.1429	0.0741	1.0741	0.1154	1.3227	0.2432
**Glucose/Lactate**	NA	0.9265	0.7486	0.5941	0.9659	0.2465	0.1437	0.3104	0.0342	0.2479	0.0565	0.2032	0.0196	0.4682	0.2104

One Way ANOVA with GraphPad Prism was performed with Tukey correction for multiple comparison. P-values < 0.05 are considered significant. Bolded numbers identify significant p values. NA, Not Applicable.

**Figure 3:**
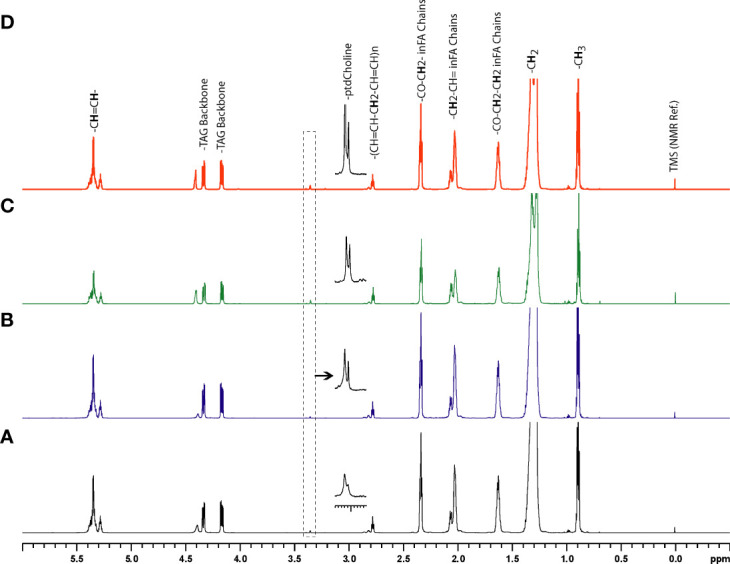
Representative ^1^H MR spectra showing metabolic differences in lipid-phase extracts of tumor tissue obtained from **(A)** normal fat, **(B)** benign lipoma (BL), **(C)** atypical lipomatous tumor (ALT), and **(D)** dedifferentiated liposarcoma (DDL).

**Figure 4:**
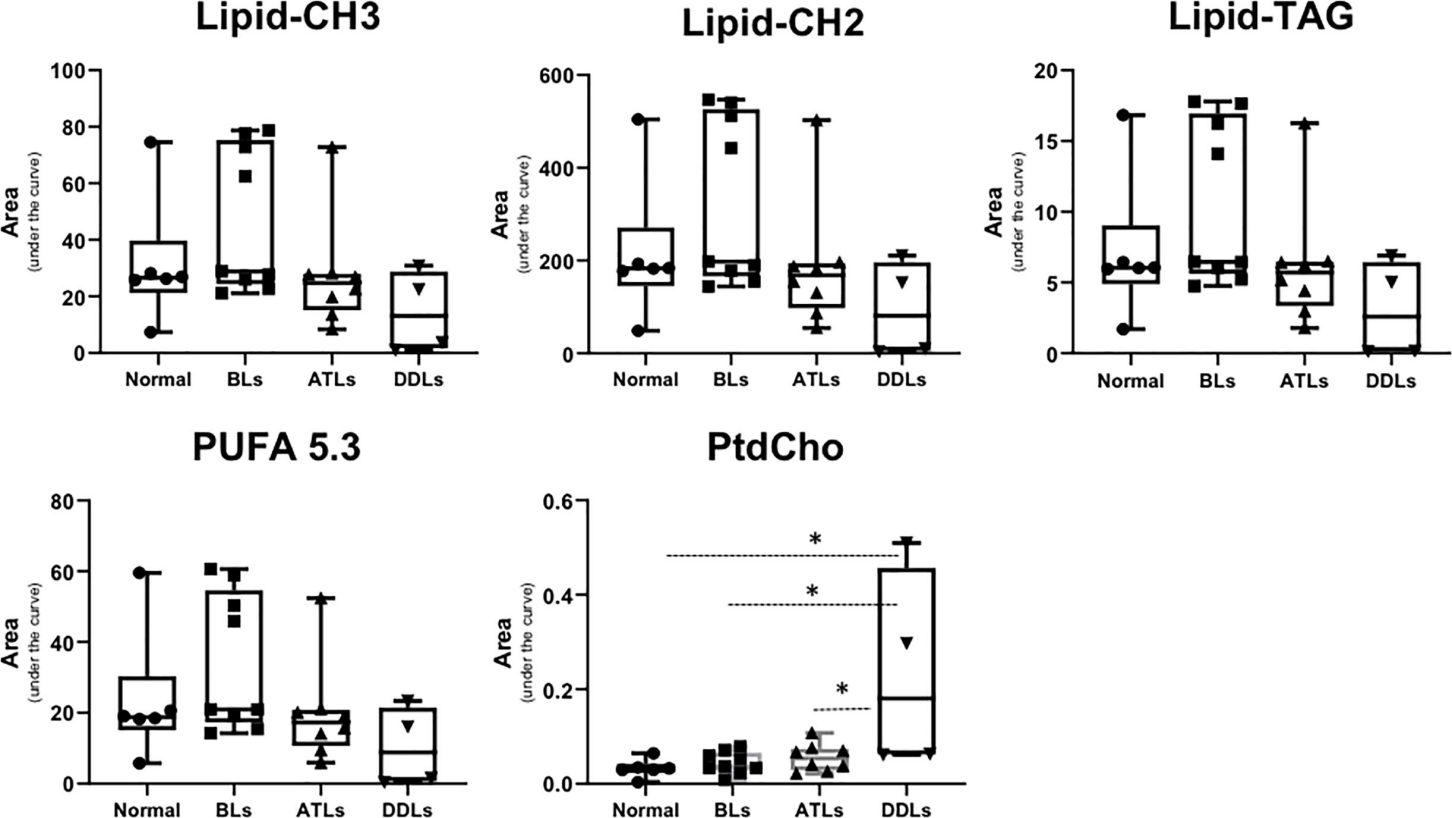
Bar plots showing semi-quantitative analysis of lipid metabolites obtained from organic fraction of normal fat tissue (n = 9), benign lipoma (BLs, n = 10), atypical lipomatous tumor (ALTs, n = 7), and dedifferentiated liposarcoma (DDLs, n = 8). Values represent mean± SE. Lipid-CH3; peak originate from –CH3 groups from all lipids at 0.9 ppm, Lipid-CH2; -CH2- group from total lipid at 1.3 ppm, Lipid-TAG; glycerol backbone of TAG lipids at 4.15 ppm, polyunsaturated fatty acids peak at 5.3 ppm and phosphatidylcholine (PtdCho) at 3.25 ppm. Values represent Mean ± SE. *P-values <0.05 are considered significant.

Representative ^1^H MR spectra of the lipid fraction obtained from dual phase extraction are presented in **Figure 3**, with the spectra normalized to the artificial signal. Lipid signals at 0.9 ppm (-CH3 group), 1.3ppm (-CH2- group), 4.15ppm (glycerol backbone of TAG), 5.30ppm PUFA (polyunsaturated fatty acids) and phosphatidylcholine (PtdCho) at 3.25ppm are presented in the bar plot in **Figure 4**. There was a trend towards an increase of all lipid peaks in BLs compared to normal tissue, and a depletion of lipid peaks in ALTs and DDLs. However, these changes were not significant in any group comparison. PtdCho levels in DDLs tumors were significantly (>0.01) increased compared to normal tissue, BLs and ALTs.

### Principal component analysis

**Figure 5:**
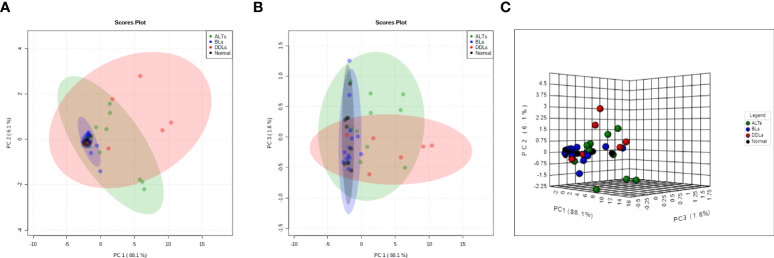
Score plots derived from principal component analysis (PCA) of the MR spectra. Two-dimensional PCA representation of the score plot showing differential clustering of each group. Two-dimensional score plot **(A)** PC1 vs PC2, **(B)** PC1 vs PC3 and **(C)** three-dimensional score plot. PC1 vs PC2 vs PC3.

To evaluate whether each group (normal fat, BLs ALTs, DDLs) could be specifically defined by metabolic profiles, a PCA analysis was performed on quantitative data obtained from the aqueous-phase spectra. Integration of the peak areas of non-overlapping aqueous-phase metabolite resonances from the ^1^H MRS spectra were acquired from a variation-size binning analysis with the water resonance excluded. The scatter plot in **Figure 5** represents the first three principal components (PC1, PC2, & PC3) generated from the PCA analysis. The plots reveal that the tumor metabolite data from normal fat and BLs are clearly separated into distinct differential clusters from ALTs and DDLs, based on distinct metabolic signatures. Clustering of normal fat with BLs, reflects the similarities in the metabolic profile between normal fat and BLs, consistent with observations that MDM2 amplification is present in ALTs and DDLs, but absent in BLs and normal tissue. Loading plots showing the metabolites playing a role in PCA clustering of each group are presented in **Supplementary Figure 3**.

## Discussion

Deficiencies in the characterization of lipomatous tumors using MRI and clinical features arise from the overlapping features of benign lipoma variants (BLs) and intermediate grade (ADL/WDLs) tumors, and to a lesser extent ADLs/WDLs and malignant tumors (DDLs). As such, histologic characterization is frequently necessary, including molecular analysis to identify amplification of MDM2, which is characteristic of ALTs/WDLs and DDLs and absent in BLs. In this study, metabolic characterization of lipomatous tumors was achieved with high-resolution ^1^H MRS, showing distinctive metabolic signatures of normal subcutaneous fat, BLs, ALT/WDLs and DDLs.

Proton MRS can be successfully incorporated into clinical scanning and has been utilized in the musculoskeletal system for tumor characterization, for both bone and soft tissue tumor types at 3T (14) and 1.5T (23, 24). Several of the metabolites such as glutamine, glutamate, lactate and alanine that were significantly altered in ADLs and DDLs can be detected by spectroscopic imaging. Based on the differences in glucose and lactate observed in our study, ^13^C spectroscopic imaging of dynamic nuclear polarization (DNP)-hyperpolarized 1-^13^C-labeled pyruvate may also be useful in characterizing lipomatous tumors. Metabolomics using high-resolution MRS is an extension of clinical scanning and a promising emerging technique for characterization of tumors (25–27). In the musculoskeletal system, metabolomics from serum and tissue samples has been previously investigated, yielding molecular signatures useful for diagnosis (28), tumor progression (29) and prognosis (30, 31). Our study utilized surgical specimens for the examination of a unique clinical problem in musculoskeletal tumor characterization: the assessment of low, intermediate and high grade lipomatous tumors. Conceivably, metabolomics may become an adjunct technique to characterize tumors following percutaneous biopsy of an indeterminate lesion (the current standard of care), and assist in directing the diagnosis toward the correct grade of tumor, an important advance for the clinical management of these lipomatous tumors. This will, however, require FDA approval for routine clinical diagnostic use.

The results of our lipid content and composition analysis share some similarities and differences with previous studies utilizing MRS to evaluate lipomatous tumors *ex vivo*. Whereas we identified a trend toward depletion of lipid peaks in ALTs/WDLs and DDLs compared to BLs, prior proton-decoupled ^13^C NMR analysis found that DDLs had diminished lipid content compared to WDLs, but contrastingly found that WDLs had increased lipid content compared to BLs (32). Additionally, previous ^1^H NMR lipid analysis by Millis *et al.* demonstrated increased PtdCho in DDLs compared to WDLs and BLs, consistent with our findings and representative of increased tissue cellularity (33). However, they also reported a twofold increase in PtdCho among WDLs compared with BLs, which our study does not support. These differences may be due to the restriction of the present study to radiologically-indeterminate tumors, likely increasing the similarities of composition between the BL and ALT/WDL groups.

In contrast, our aqueous-phase metabolite data show that ALT/WDLs have a metabolic profile more similar to DDLs than to BLs. In addition to providing an adjunct diagnostic tool, these findings may help advance understanding of liposarcomagenesis. Although both ALT/WDLs and DDLs are characterized by MDM2 and CKD4 amplification, co-overexpression of these oncogenes in human bone marrow stem cells with additional oncogenic hits yields a DDL-like morphology (34). Thus, DDL transformation within existing ALT/WDLs remains poorly understood. However, recent *in vivo* experiments have elucidated the p53-independent role of MDM2 as a regulator of amino acid metabolism and redox homeostasis (35). Additionally, higher levels of MDM2 amplification in DDL cell lines were associated with metabolic perturbations including increased sphingolipid metabolism and *de novo* fatty acid synthesis (36). Corroborating these findings, we report amino acid alterations that characterize ALT/WDLs and DDLs, but not BLs. Additionally, in our study DDLs were distinguished from ALT/WDLs by an elevated glucose-to-lactate ratio, supporting further investigation into therapeutic strategies targeting MDM2-mediated metabolic pathways, especially *de novo* serine synthesis (37)

The limitations of our study include the sample size, increase of which might facilitate identification of additional features which distinguish DDLs from ALT/WDLs. Additionally, we did not obtain a normal fat sample from each patient, although this may not have impacted our results as the normal fat specimens had a fairly homogenous metabolic profile that was similar to BL and distinguishable from ALT/WDL and DDL. Finally, we excluded locally recurrent cases, which may represent the ALT/WDLs most likely to progress to DDL. However, a recent attempt to distinguish recurrent from primary ALT/WDL by comparing microRNA expression and DNA methylation found no clear distinctions (38). Despite these limitations, our study presents high-resolution ^1^H MRS *ex vivo* characterization of low, intermediate, and high grade lipomatous tumors.

In conclusion, ^1^H MRS demonstrates distinct metabolic signatures of BLs, ALT/WDLs and DDLs. Further development of these metabolic profiles could lead to the incorporation of noninvasive *in vivo* and *ex vivo* MRS into clinical scanning as a clinically-important, lipomatous tumor characterization tool. Additionally, our study supports future research into liposarcoma therapies targeting MDM2-mediated metabolic pathways which enable cells to grow despite oxidative and nutrient stress.

## Data availability statement

The raw data supporting the conclusions of this article will be made available by the authors, without undue reservation.

## Ethics statement

The studies involving human participants were reviewed and approved by Johns Hopkins IRB. The patients/participants provided their written informed consent to participate in this study.

## Author contributions

All authors listed have made a substantial, direct, and intellectual contribution to the work and approved it for publication.

## Funding

Support from NIH R35 CA209960, R01 CA82337, R01 CA253617 and R01 CA193365 is gratefully acknowledged.

## Conflict of interest

The authors declare that the research was conducted in the absence of any commercial or financial relationships that could be construed as a potential conflict of interest.

## Publisher’s note

All claims expressed in this article are solely those of the authors and do not necessarily represent those of their affiliated organizations, or those of the publisher, the editors and the reviewers. Any product that may be evaluated in this article, or claim that may be made by its manufacturer, is not guaranteed or endorsed by the publisher.
