# Supplementary material for: Characterization of lipomatous tumors with high-resolution 1H MRS at 17.6T: Do benign lipomas, atypical lipomatous tumors and liposarcomas have a distinct metabolic signature?
Source: Front Oncol. 2022 Sep 9;12:920560. doi: 10.3389/fonc.2022.920560 (PMC9500232; doi:10.3389/fonc.2022.920560)

Supplementary Figures

Characterization of Lipomatous Tumors with High-resolution ^{1}H MRS at 17.6T: Do Benign Lipomas, Atypical Lipomatous Tumors and Liposarcomas Have a Distinct Metabolic Signature?

Santosh Kumar Bharti, PhD [1‡], Brett A. Shannon, MD[2‡], Raj Kumar Sharma, PhD[1], Adam S. Levin, MD[2], Carol D. Morris, MD, MS[2], Zaver M. Bhujwalla, PhD*[1,3,4], Laura M. Fayad, MD, MS*[2,5]

[1]Division of Cancer Imaging Research, The Russell H. Morgan Department of Radiology and Radiological Science; [2]Department of Orthopaedic Surgery; [3]Sidney Kimmel Comprehensive Cancer Center; [4]Department of Radiation Oncology and Molecular Radiation Sciences; [5]Musculoskeletal Radiology, The Russell H. Morgan Department of Radiology and Radiological Science; The Johns Hopkins University School of Medicine, Baltimore, MD.

Running Title: Proton MRS demonstrates distinct metabolic signatures of lipomatous tumors

Keywords: Lipoma, Liposarcoma, Atypical Lipomatous Tumor, ^{1}H MR spectroscopy, metabolites

‡These investigators contributed equally to this work and have equal first authorship.

*****Equal senior authorship

Conflict of Interest: The authors disclose no potential conflicts of interest.

Funding Statement: Support from NIH R35 CA209960, R01 CA82337, R01 CA253617 and R01 CA193365 is gratefully acknowledged.

Figure Legends

Supplementary Figure 1: Representative ^{1}H MR spectra with the TSP signal included, showing metabolic differences in aqueous-phase extracts of tumor tissue obtained from (A) normal fat, (B) benign lipoma, (C) atypical lipomatous tumor, and (D) dedifferentiated liposarcoma. Normal fat tissues are non-involved tissues obtained during tumor excision surgery.

Supplementary Figure 2: Bar plots showing 1H MR spectroscopic analysis of aqueous-phase tumor metabolites obtained from normal fat (n=9), benign lipoma (BLs, n=14), atypical lipomatous tumor (ALTs, n=11), and dedifferentiated liposarcoma (DDLs, n=6). Values represent mean± std error of mean (SE). Abbreviations: BCAA, branch chain amino acids; BHB, beta-hydroxybutyrate. Statistical analysis for the different groups can be found in Table 2.

Supplementary Figure 3: Loading plots derived from principal component analysis (PCA) of the MR spectra. One-dimensional PCA representation of the loading plot showing the metabolites playing a role in PCA clustering of each group.

Supplementary Figure 1: Representative ^{1}H MR spectra with the TSP signal included, showing metabolic differences in aqueous-phase extracts of tumor tissue obtained from (A) normal fat, (B) benign lipoma, (C) atypical lipomatous tumor, and (D) dedifferentiated liposarcoma. Normal fat tissues are non-involved tissues obtained during tumor excision surgery.

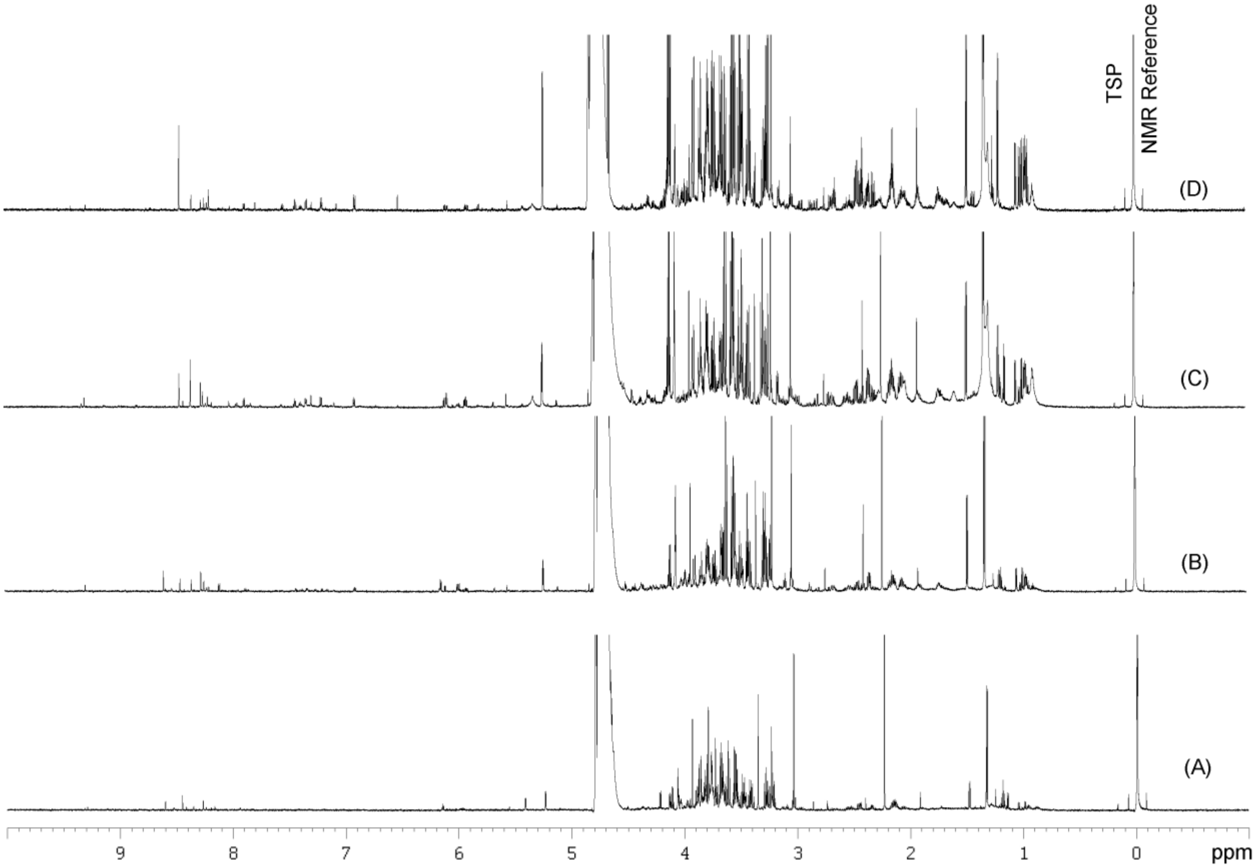

Supplementary Figure 2: Bar plots showing 1H MR spectroscopic analysis of aqueous-phase tumor metabolites obtained from normal fat (n=9), benign lipoma (BLs, n=14), atypical lipomatous tumor (ALTs, n=11), and dedifferentiated liposarcoma (DDLs, n=6). Values represent mean± std error of mean (SE). Abbreviations: BCAA, branch chain amino acids; BHB, beta-hydroxybutyrate. Statistical analysis for the different groups can be found in Table 2.

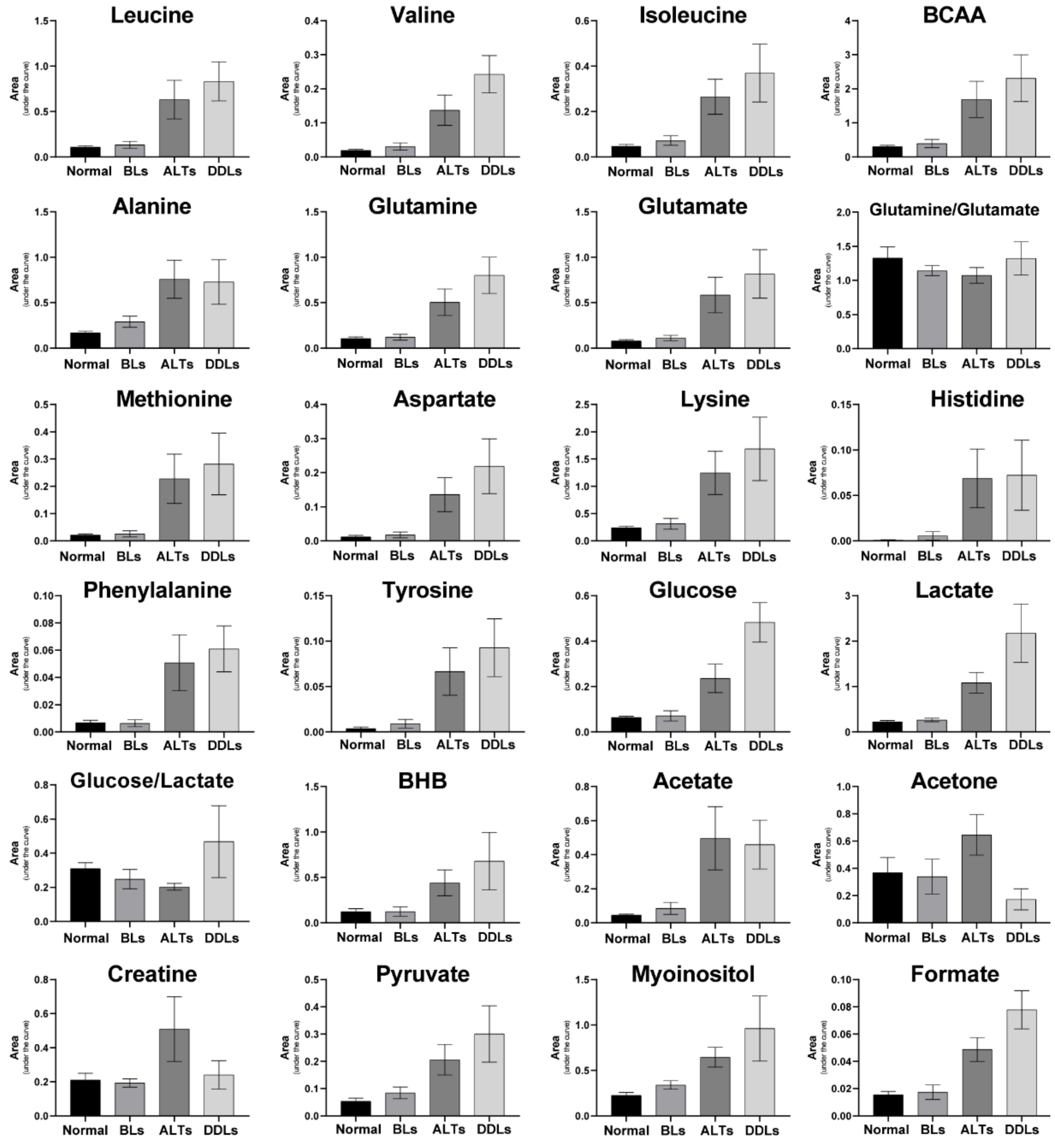

Supplementary Figure 3: Loading plots derived from principal component analysis (PCA) of the MR spectra. One-dimensional PCA representation of the loading plot showing the metabolites playing role in PCA clustering of each group.

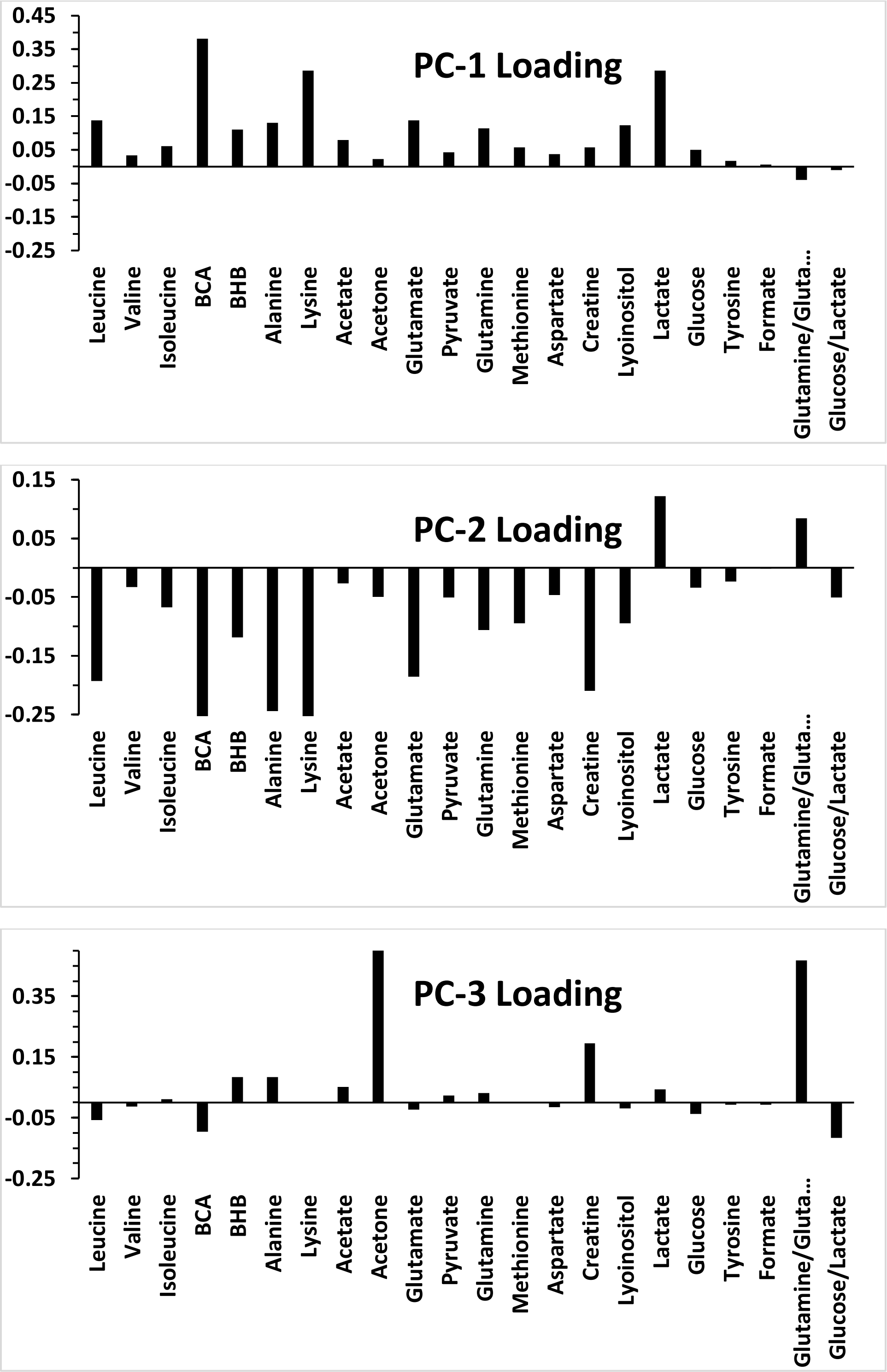

Supplement: Supplementary file 1 [file DataSheet_1.pdf]
